# A140 DUPILUMAB IMPROVED HISTOLOGY, CLINICAL SYMPTOMS, AND ENDOSCOPIC FEATURES IN ADULTS AND ADOLESCENTS WITH EOSINOPHILIC ESOPHAGITIS THROUGH 52 WEEKS REGARDLESS OF BASELINE DISEASE DURATION: POST-HOC SUBGROUP ANALYSIS OF THE PHASE 3 LIBERTY EOE TREET STUDY

**DOI:** 10.1093/jcag/gwae059.140

**Published:** 2025-02-10

**Authors:** N Gonsalves, C Schlag, C Santander, K Peterson, R Alkhouri, C Xia, B P Raphael, A Radwan

**Affiliations:** Gastroenterology, Northwestern University Feinberg School of Medicine, Chicago, IL; University Hospital Zürich, Zürich, Switzerland; Hospital Universitario de La Princesa, Madrid, Spain; University of Utah, Salt Lake City, UT; Sanofi, North York, ON, Canada; Regeneron Pharmaceuticals Inc., Tarrytown, NY; Regeneron Pharmaceuticals Inc., Tarrytown, NY; Regeneron Pharmaceuticals Inc., Tarrytown, NY

## Abstract

**Background:**

Limited literature is available on EoE disease duration and response to therapy. Dupilumab is approved for EoE in patients aged ≥12 years, weighing ≥40 kg, in the EU, and in patients aged ≥1 year, weighing ≥15 kg, in the USA and Canada.

**Aims:**

To assess the long-term efficacy of dupilumab in patients with EoE stratified by baseline disease duration.

**Methods:**

In Part B of LIBERTY EoE TREET (NCT03633617), patients received 24 weeks of dupilumab 300 mg or placebo weekly (qw). Eligible patients who completed Part B received dupilumab through 52 weeks (Part C). Co-primary endpoints were the proportion achieving peak esophageal intraepithelial eosinophil count (PEC) ≤6 eosinophils/high-power field (eos/hpf) and absolute change in Dysphagia Symptom Questionnaire (DSQ) total score. Secondary endpoints included proportion achieving PEC <15 eos/hpf, percent change in PEC, and mean change in Endoscopic Reference Score (EREFS) total score and Histologic Scoring System (HSS) grade/stage scores. Data were stratified by baseline disease duration defined as time since diagnosis (≤5 years, >5-≤10 years, >10 years).

**Results:**

At Part B baseline in the dupilumab and placebo groups, respectively, 43 and 48 patients had EoE for ≤5 years, 22 and 13 patients had EoE for >5-≤10 years, and 15 and 18 patients had EoE for >10 years. At Week 24, PEC ≤6 eos/hpf and DSQ total score favored dupilumab over placebo, regardless of disease duration. Dupilumab also improved percent change in PEC and change in EREFS and HSS grade/stage scores across all disease duration subgroups vs placebo (**Table**). At Week 52, improvements were maintained in patients who received continued dupilumab; patients who switched from placebo to dupilumab at Week 24 also showed improvement in all outcomes. Overall safety was consistent with the known dupilumab safety profile.

**Conclusions:**

These results show that dupilumab 300 mg qw is an effective long-term treatment for patients with EoE, including a subset of patients with longstanding disease potentially inadequately addressed by alternative therapies.

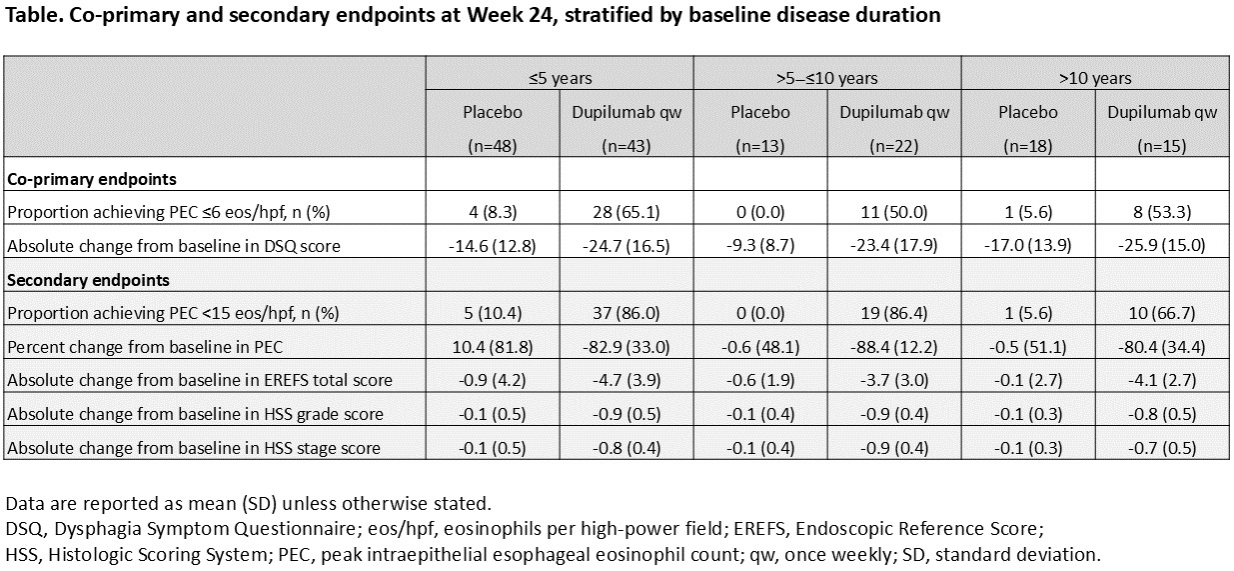

**Funding Agencies:**

Research sponsored by Sanofi and Regeneron Pharmaceuticals Inc.

